# Explaining SCAN connectivity and hyperconnectivity through psychological theories of perception-action binding

**DOI:** 10.3389/fnhum.2026.1808533

**Published:** 2026-05-29

**Authors:** Bernhard Pastötter

**Affiliations:** Department of Psychology, Trier University, Trier, Germany

**Keywords:** beta-band synchronization, Parkinson's disease, perception-action binding, Somato-Cognitive Action Network (SCAN), Theory of Event Coding (TEC)

Here, I propose a psychological account of connectivity within the Somato-Cognitive Action Network (SCAN; Gordon et al., 2023) grounded in established theories of perception-action binding. Specifically, I argue that excessive coupling in Parkinson's disease may partially reflect pathological hypermaintenance of event files rather than merely stronger action representations. Recent studies have identified a brain network that integrates goals, bodily signals, and movement, and have shown that this network becomes excessively coupled in Parkinson's disease. Established psychological theories of how perception and action interact provide a functional explanation for why such overly stable coupling may reduce behavioral flexibility and contribute to motor rigidity.

Recently, Gordon et al. (2023) reshaped the classical view of primary motor cortex by identifying the SCAN interleaved with effector-specific motor zones. Rather than forming a continuous homunculus, foot, hand, and mouth representations are punctuated by distinct inter-effector regions that show strong mutual connectivity and preferential coupling to cingulo-opercular control systems. These territories lack strict movement specificity and are consistently recruited during action planning, coordination, and whole-body control. Whereas effector-specific regions appear to code relatively isolated movement components (e.g., hand, foot, or mouth actions), the inter-effector regions may support the integration of multiple action-relevant features across effectors. Rather than representing single movements, these regions may combine bodily states, goals, and movement components into unified perception-action representations. The resulting architecture moves beyond a purely execution-based view of motor cortex and instead highlights cortical regions specialized for integrating cognitive, autonomic, and bodily signals. Functionally, the SCAN appears ideally positioned to support perception-action binding.

Building on this anatomical framework, Ren et al. (2026) demonstrated that Parkinson's disease is characterized by increased cortico-subcortical functional connectivity within the SCAN. Across multiple interventions—including dopaminergic medication, deep-brain stimulation, transcranial magnetic stimulation, and magnetic resonance imaging-guided focused ultrasound—clinical improvement was accompanied by normalization of this coupling. These findings localize disease-related abnormalities to a circuit specifically implicated in action integration. They also raise a functional question: why should increased connectivity within an integrative perception-action network manifest as rigidity and reduced flexibility?

Long-standing psychological theory offers a direct answer. For more than a century, ideomotor theory has proposed that perception and action rely on shared representational codes (James, 1890; Greenwald, 1970). Actions are selected by anticipating their sensory consequences, implying that voluntary behavior emerges from integrated perception-action states rather than independent motor commands (Prinz, 1997; Hommel et al., 2001). This principle of common coding has since become foundational for contemporary models of action control (Hommel, 2004; Frings et al., 2020).

The Theory of Event Coding (TEC; Hommel et al., 2001) formalizes this idea by proposing that stimuli, responses, and internal states—including goals, anticipated action effects, and task sets—are temporarily bound into unified event files, which serve as the basic units of behavior. Binding and Retrieval in Action Control (BRAC; Frings et al., 2020) further specifies the dynamics of these units by distinguishing between binding, maintenance, and retrieval processes. Crucially, these models emphasize a functional trade-off: adaptive behavior requires stability, but also rapid updating. Event files must persist long enough to coordinate action yet dissolve quickly when circumstances change.

Accordingly, event files are conceived as short-lived representational structures. They typically persist for only seconds and decay unless reactivated, allowing efficient short-term reuse of recent action plans. Such transient bindings should not be equated with durable learning of stimulus-response associations; whether and how temporary bindings consolidate into longer-term memory traces remains an open question with clear boundary conditions (Frings et al., 2024b). Stability is therefore adaptive only within limits. Flexible behavior additionally requires that outdated bindings can be released and new ones formed.

This perspective also clarifies when integration becomes maladaptive. If maintenance dominates and previously formed bindings persist beyond their useful time window, updating is impaired. Such hyperpersistence—or hyperbinding—predicts behavioral rigidity, slowed initiation, and perseveration. Viewed through this lens, the Parkinson's disease findings by Ren et al. (2026) acquire a straightforward interpretation. Because the SCAN is anatomically organized to integrate bodily state, goals, and movement, increased cortico-subcortical connectivity within this circuit is consistent with overly persistent perception-action bindings. If event files cannot be readily released, previously established action states may continue to dominate behavior. Bradykinesia, rigidity, and impaired initiation can thus be understood not only as deficits of motor execution but as failures to disengage from entrenched event-file states.

Notably, reductions in SCAN hyperconnectivity were accompanied by clinical improvement under dopaminergic medication and neuromodulation in the Ren et al. (2026) study, indicating that coupling within this network is behaviorally relevant rather than epiphenomenal. What remains less explicit is the physiological mechanism through which altered connectivity translates into reduced behavioral flexibility. Psychological and neurocomputational models of perception-action binding offer a mechanistic explanation for this link.

An important neural bridge between these psychological accounts and the SCAN may be provided by the action observation network (AON). The AON, encompassing posterior superior temporal, inferior parietal, and inferior frontal regions, is widely considered the neural substrate through which perception and action share common representational codes in action observation (Molenberghs and Cunnington, 2012; Thompson and Bird, 2019). Recent work further shows that this network is dynamically configured according to the observer's goals: focusing on how an action is performed vs. why it is performed selectively alters connectivity within the AON (Zhou et al., 2023). Likewise, posterior temporal regions respond more strongly when attention is directed toward body kinematics than toward actor identity or object goals (Zhou et al., 2023). These findings closely parallel TEC and BRAC, suggesting that perception-action bindings are flexibly weighted according to current goals and task demands. In this view, the SCAN may constitute a large-scale extension of the AON into inter-effector motor cortex, providing the neural substrate through which bodily states, goals, and movement plans become integrated into temporary event files. The question remains how the persistence of these states is implemented physiologically.

Converging electrophysiological evidence supports this account. Excessive maintenance of motor states is consistently indexed by increased beta-band synchronization, a signature commonly linked to the stabilization of ongoing sensorimotor representations (Engel, 2010). Importantly, studies in healthy participants show that post-response beta dynamics scale with the persistence of short-term event files: individuals exhibiting larger beta synchronization display stronger stimulus-response binding effects and slower disintegration of recently formed stimulus-response compounds, indicating prolonged maintenance of event files (Pastötter et al., 2021). Extending this logic to clinical populations, work applying BRAC to functional movement disorders demonstrated enhanced post-movement beta synchronization consistent with abnormally persistent stimulus-response bindings (Pastötter et al., 2024). Together, these findings suggest that beta oscillations provide a neural index of event-file maintenance, such that excessive synchronization reflects pathological hypermaintenance. Similar mechanisms may generalize across movement disorders, including Parkinson's disease (Meppelink and de Jong, 2024).

The proposed account can be further specified within Friston's predictive coding and active inference framework (Friston, 2005, 2010). From this perspective, the currently active event file can be understood as a sensorimotor prior specifying the expected bodily state, movement, and action consequences. Adaptive behavior requires a balance between the currently active prior and incoming sensory prediction errors, such that priors can be updated when new evidence indicates that a different action state has become more appropriate. Beta-band synchronization may index the precision-weighting of the current sensorimotor prior—that is, the confidence assigned to the presently active event file. Excessive beta synchronization and SCAN hyperconnectivity in Parkinson's disease would therefore reflect an over-weighting of the current perception-action state. Because the active prior is assigned excessive precision, sensory prediction errors have insufficient impact to trigger updating, leaving the system locked into an entrenched perception-action state. Bradykinesia and rigidity may thus arise because previously established event files cannot be flexibly disengaged.

Indeed, Parkinson's disease is consistently characterized by elevated beta-band synchronization throughout cortico-basal-ganglia motor circuits, with beta power scaling with bradykinesia and rigidity and normalizing under dopaminergic treatment or deep-brain stimulation (Kühn et al., 2006; Sharott et al., 2014; Little, 2014). If the SCAN provides the cortical substrate for perception-action integration, its hyperconnectivity may covary with these beta dynamics. SCAN coupling (systems level), post-response beta persistence (oscillatory level), and partial-repetition costs (behavioral level)—that is, slower and less efficient performance when some features (e.g., stimulus features) of a previously established event file repeat whereas others (e.g., response features) must be updated (Frings et al., 2024a)—may thus constitute converging markers of a common mechanism: adaptive stability in healthy controls but overly persistent event files that impair flexible behavior in patient groups with specific movement disorders.

Taken together, the studies by Gordon et al. (2023) and Ren et al. (2026) identify the SCAN as a neural substrate for integrating goals, physiology, and movement, and demonstrate its disruption in disease. Established psychological theories provide a complementary functional account of how such integration shapes behavior and why excessive coupling becomes detrimental. Ideomotor theory, TEC, and BRAC thus offer a mature theoretical language that directly links neural integration to observable symptoms. The AON and SCAN may provide the neuroanatomical substrate of these processes, whereas predictive coding and active inference specify why adaptive event-file maintenance can become pathological hypermaintenance.

Considering these psychological foundations may therefore clarify the functional significance of SCAN hyperconnectivity in Parkinson's disease and related disorders. Together, these converging perspectives suggest that pathological SCAN hyperconnectivity may reflect a failure to flexibly update perception-action states, thereby linking neural coupling, beta-band synchronization, and behavioral rigidity within a common framework.
